# Supplementary material for: Highly efficient and air-stable Eu(II)-containing azacryptates ready for organic light-emitting diodes
Source: Nat Commun. 2020 Oct 15;11:5218. doi: 10.1038/s41467-020-19027-x (PMC7562750; doi:10.1038/s41467-020-19027-x)
Supplement: Supplementary file 1 — Supplementary Information [file 41467_2020_19027_MOESM1_ESM.pdf]

Supplementary Information

Highly Efficient and Air-Stable Eu(II)-Containing Azacryptates Ready for Organic Light-Emitting Diodes

Jiayi Li, Liding Wang, Zifeng Zhao, Boxun Sun, Ge Zhan, Huanyu Liu, Zuqiang Bian, and *Zhiwei Liu*

Photophysical measurement

UV-vis absorption spectra were obtained from a Shimadzu UV-3100 spectrometer. Steady/transient PL spectra were recorded on an Edinburgh Analytical Instruments FLS980 spectrophotometer, equipped with pulsed laser (Edinburgh Ltd. Co.). The PLQYs of crystalline powder were measured by an absolute PLQY measurement system on C9920-02 from Hamamatsu Company. The transient PL data is processed using OriginPro 2020b (academic version). The datapoints were shifted to make sure that t_0 corresponds to the maximum intensity (I_0). The y axis is using natural logarithmic coordinate. For an ideal decay process without other competing path, the luminescent intensity will undergo exponential decay.

$$I(t) = I_0 e^{-\frac{t}{\tau}}$$

$$\ln[I(t)] = Const. - \tau^{-1} t$$

Thermal properties measurements

Thermogravimetric analyses were recorded with a Q600SDT instrument with a heating rate of 15 °C min^{-1} from room temperature to 700 °C under fixed N_2 flow.

Buried volume calculation

The calculations were conducted in the web application named SambVca (https://www.molnac.unisa.it/OMtools/sambvca2.1/index.html). The input file is the xyz file of each complex. The top of the N_8 ligand, Eu^{2+} center and hydrogens are omitted in the calculation as illustrated from **Fig. S7(a)** to **7(b)**. The coordinate system is established, where Eu^{2+} being the coordinate origin.

Density functional theory (DFT) calculations

All calculations were performed with the ORCA program package.[1] For ground state geometry optimizations, the hybrid B3LYP[2-5] density functional was used without symmetry constraints. Def2-ECP pseudopotential[6] with Def2-TZVP valence basis sets were used for Eu (28 core electrons). The all-electron triple-ζ quality Def2-TZVP basis[7] sets were applied for the remaining elements in these compounds. We had included the atom-pairwise dispersion correction with Becke-Johnson damping (D3BJ) to account for the van der Waals interaction.[8-11] In the single-point time-dependent DFT (TD-DFT) calculation, the PBE functional was applied with Def2-SV(P) bases sets[12] for all elements (Def2-ECP pseudopotential was also applied for Eu). The RI plus chain of spheres (RIJCOSX) approximation[13] was used to accelerate all the calculations with the Weigend's "universal" Coulomb fitting auxiliary basis set def2/J.[14]

HOMO/LUMO level calculations

Ultraviolet photoelectron spectroscopy was measured on AXIS Supra X-ray photoelectron spectrometer from Kratos Analytical Ltd. Based on the data deduced from the ultraviolet photoelectron spectra and the edge of ultraviolet absorption spectra (λ = 527 nm), the HOMO and LUMO energy levels of the two complexes are calculated as follows:

$EuBr_2$-N_8:

$E_{HOMO} = -(h\nu - E_B + E_A) = -(21.22\ eV - 18.86\ eV + 3.19\ eV) \approx -5.6\ eV$

$E_{LUMO} = E_{HOMO} + hc/\lambda = -5.6\ eV + 1240/527\ eV \approx -3.2\ eV$

EuI_2-N_8:

$E_{HOMO} = -(h\nu - E_B + E_A) = -(21.22\ eV - 17.70\ eV + 2.15\ eV) \approx -5.7\ eV$

$E_{LUMO} = E_{HOMO} + hc/\lambda = -5.7\ eV + 1240/527\ eV \approx -3.3\ eV$

OLEDs fabrication and measurements

Indium tin oxide (ITO) patterned anode was commercially available with a sheet resistance of 14 Ω $square^{-1}$ and 80 nm thickness. Before fabrication, the patterned indium tin oxide (ITO) substrate is cleaned with deionized water, acetone, and ethanol. The organic and metal layers were deposited in different vacuum chambers with a base pressure better than 1×10^{-4} Pa. The thickness of each layer and the evaporation rate of all the materials were monitored using quartz crystal monitors. Rate of deposition were maintained at 0.5-1 $Å\cdot s^{-1}$ for organic materials and 2-3 $Å\cdot s^{-1}$ for cathode, respectively. The active area for each device is 4 mm^2. All electrical testing and optical measurements were performed under ambient conditions with encapsulation of devices in a glovebox. The EL spectra, current density-voltage-luminance (J-V-L) and EQE characteristics were measured by computer-controlled Keithley 2400 source meter and absolute EQE measurement system (C9920-12) with photonic multichannel analyzer (PMA-12, Hamamatsu Photonics).

Supplementary Table 1. Summary of crystallographic data of EuX_2-N_n

	$EuBr_2$-N_4·$2CH_3OH$	EuI_2-N_4	$EuBr_2$-N_8	EuI_2N_8·CH_3OH
Formula	$C_{18}H_{48}Br_2EuN_8O_2$	$C_{16}H_{40}EuI_2N_8$	$C_{18}H_{42}Br_2EuN_8$	$C_{19}H_{46}EuI_2N_8O$
Mw	722.42	750.32	682.37	808.40
Crystal	monoclinic	orthorhombic	orthorhombic	Orthorhombic
Space group	P21/n	Cmca	Pca21	P212121
a (Å)	10.3099(2)	14.8426(3)	15.8192(5)	10.9883(2)
b (Å)	12.7018(3)	14.6773(3)	12.1451(3)	14.0815(3)
c (Å)	11.5506(3)	12.9738(3)	13.3108(5)	18.5562(4)
α (°)	90.00	90.00	90.00	90.00
β (°)	105.094(3)	90.00	90.00	90.00
γ (°)	90.00	90.00	90.00	90.00
Volume (Å^3)	1460.42(6)	2826.33(10)	2557.35(14)	2871.23(10)
Z	2	4	4	4
T (K)	180.01(10)	180.00(10)	290.00(10)	180.00(10)
D_{calc} (g·cm^{-3})	1.638	1.763	1.772	1.870
F (000)	722.0	1444	1356	1572
θ range (°)	2.5990 - 29.0420	2.505 - 27.483	2.575 - 27.476	2.1600 –
	$-13 \le h \le 12$	$-18 \le h \le 19$	$-12 \le h \le 20$	$-9 \le h \le 14$
Index range	$-16 \le k \le 16$	$-19 \le k \le 18$	$-15 \le k \le 15$	$-18 \le k \le 18$
	$-14 \le l \le 9$	$-14 \le l \le 16$	$-11 \le l \le 17$	$-21 \le l \le 24$
GOF on F^2	1.020	1.020	1.020	1.039
R_1/wR_2	0.0217/0.0505	0.0262/0.0779	0.0305/0.0661	0.0203/0.0438
R_1/wR_2	0.0270/0.0489	0.0281/0.0792	0.0415/0.0691	0.0215/0.0442

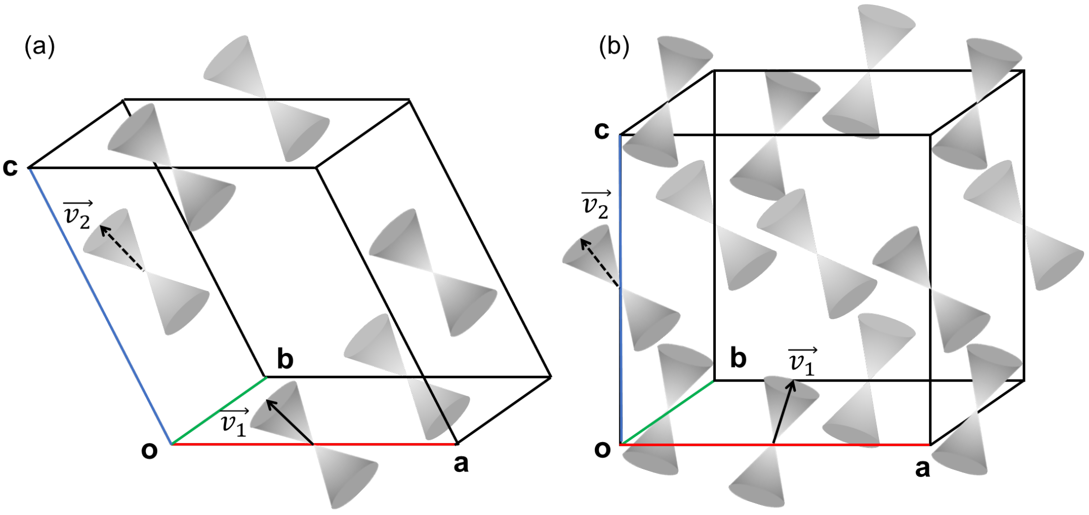

Supplementary Figure 1. The illustration of (a) $EuBr_2$-N_4 and (b) EuI_2-N_4 crystal structure. The double cone represents Eu^{2+} coordinated by two N_4 ligands. As shown in the figure, there are two sets of $[Eu(N_4)_2]^{2+}$ with different orientations in crystal. The orientation is illustrated by the unit vector, from the coordinate of Eu^{2+} to the midpoint in the upper N_4 ligand. Then the angle θ between these two vectors is calculated to compare. For $EuBr_2$-N_4: v_1=(-0.79, -0.085, 0.60), v_2=(-0.79, 0.085, 0.60), θ=9.7 °. For EuI_2-N_4: v_1=(0, 0.82, 0.56), v_2=(0, -0.83, 0.53), θ=111.0 °.

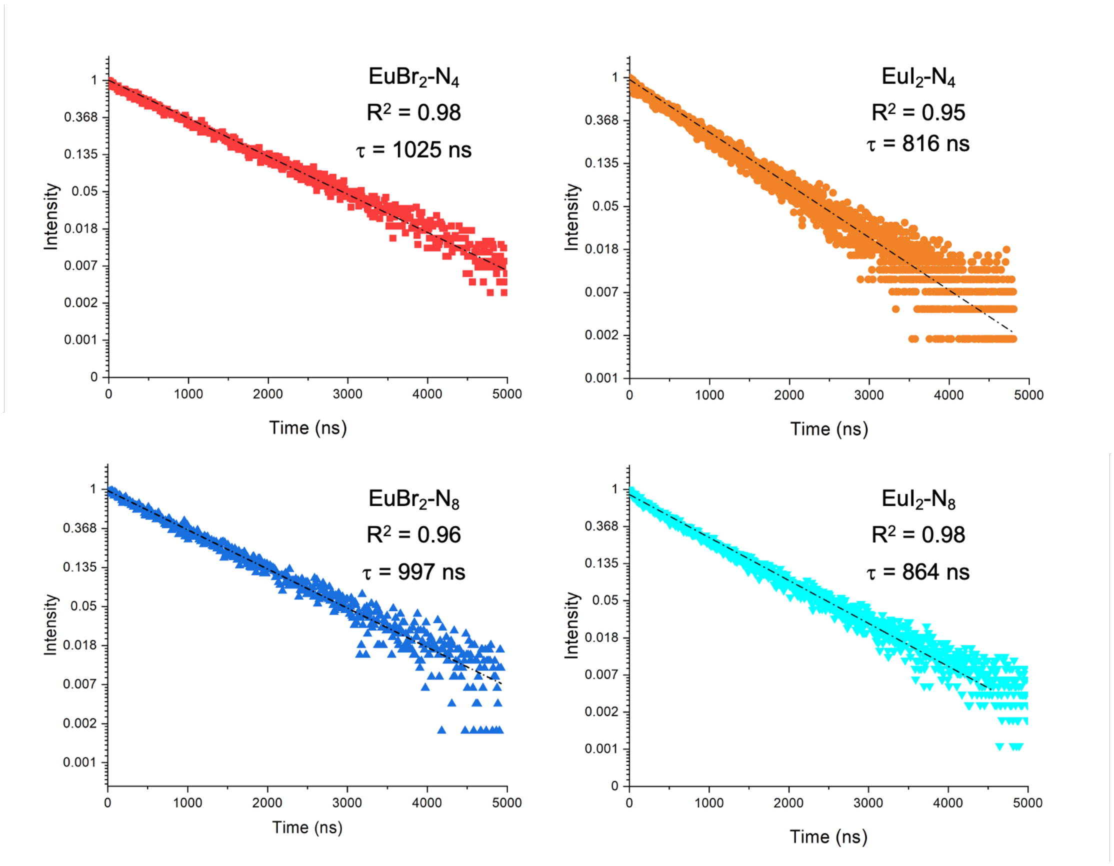

Supplementary Figure 2. Linear fit of the PL decay data.

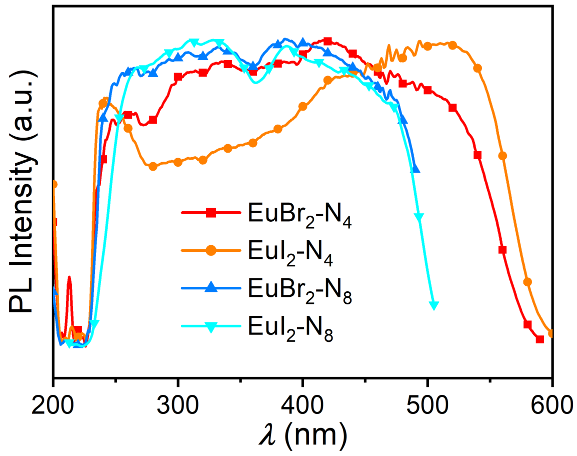

Supplementary Figure 3. Excitation spectra of EuX_2-N_n complexes. The detected wavelength is determined by the peak emission of each complex, i.e. 605, 613, 510, and 515 nm for $EuBr_2$-N_4, EuI_2-N_4, $EuBr_2$-N_8, and EuI_2-N_8, respectively.

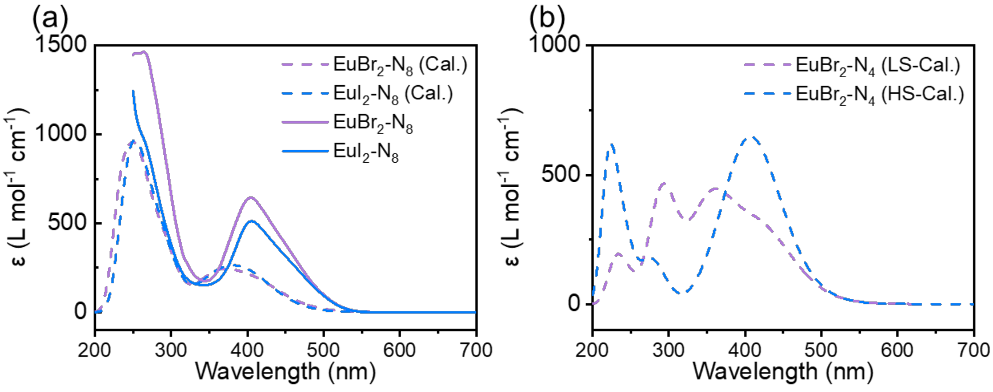

Supplementary Figure 4. (a) UV-vis spectra from experiment (solid lines) and TD-DFT calculations (dash lines) of EuX_2-N_8, (b) UV-vis spectra from TD-DFT calculations of $EuBr_2$-N_4: two possible structures of high symmetry (HS) and low symmetry (LS).

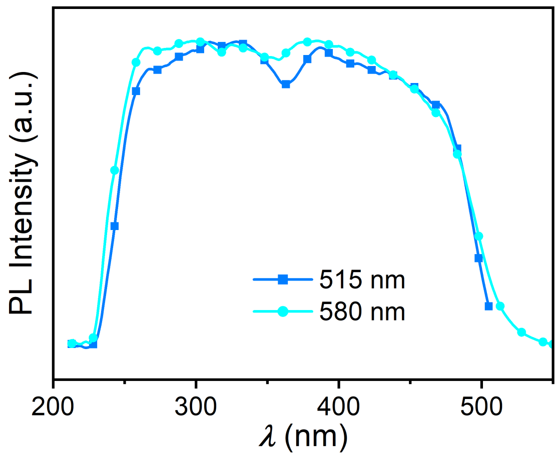

Supplementary Figure 5. The excitation spectra of ground sample of EuI_2-N_8. The 515 nm peak is the max emission of this complex while the 580 nm peak is chosen to reduce the influence of 515 nm emission.

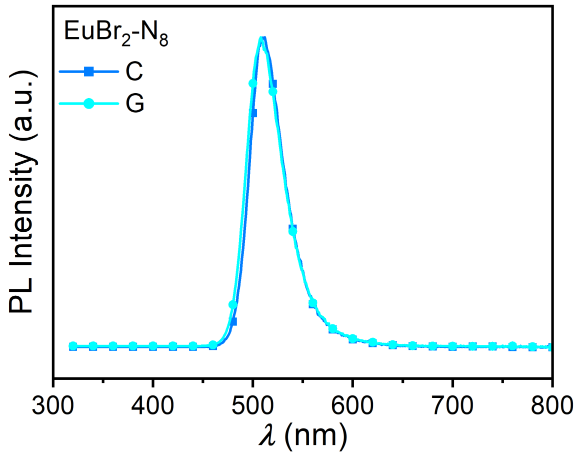

Supplementary Figure 6. The emission spectra of crystals (C) and ground samples (G) of $EuBr_2$-N_8.

Supplementary Table 2. The analysis of TGA data of four EuX_2-N_n complexes.

	$EuBr_2$-N_4	EuI_2-N_4	$EuBr_2$-N_8	EuI_2-N_8
Theoretical value[a)]	45.3	51.9	45.7	52.3
Practical value	43.7	50.7	43.13	47.2
RE[b)]	-0.035	-0.023	-0.056	-0.098

[a)] The theoretical values are calculated as: $M_{EuX2}/M_{EuX2\text{-}Nn}$. [b)] The relative errors are calculated as (practical value - theoretical value)/theoretical value.

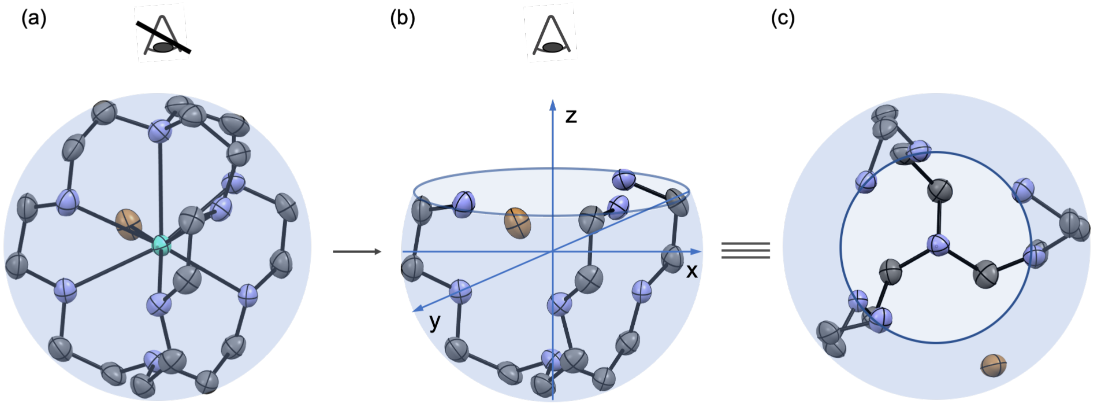

Supplementary Figure 7. The illustration of $\%V_{bur}$ calculation: (a) the crystal structure of $EuBr_2$-N_8, (b) eliminating the top of the N_8 ligand and Eu^{2+} center, establishing the coordinate system, (c) top view of (b).

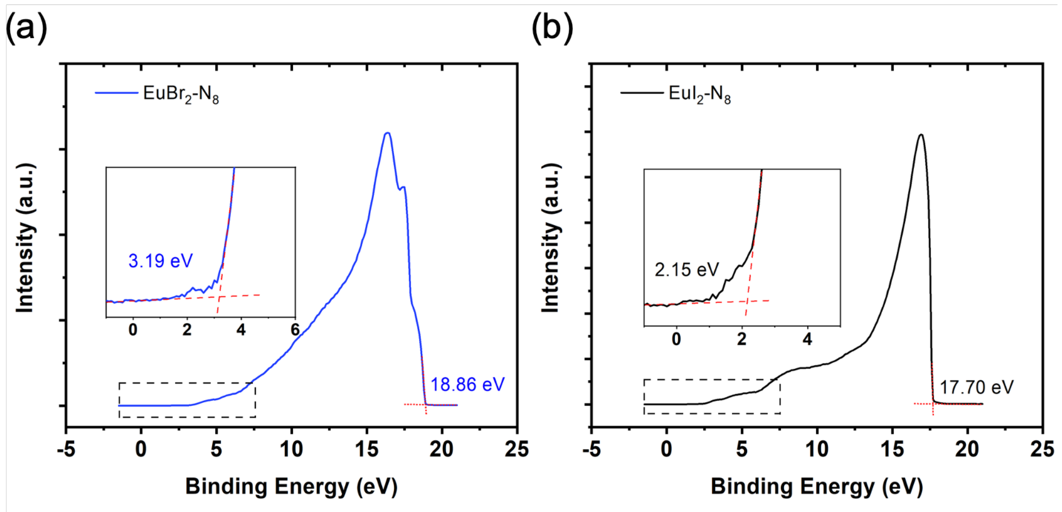

Supplementary Figure 8. The ultraviolet photoelectron spectroscopy of (a) $EuBr_2$-N_8 and (b) EuI_2-N_8.

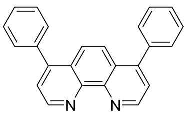

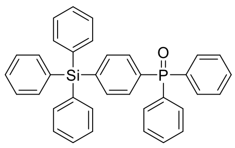

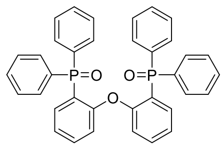

Bphen TSPO1 DPEPO

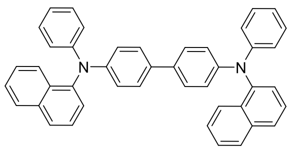

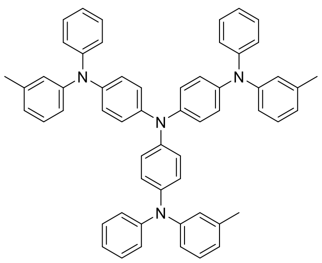

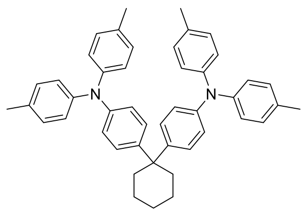

NPB m-MTDATA TAPC

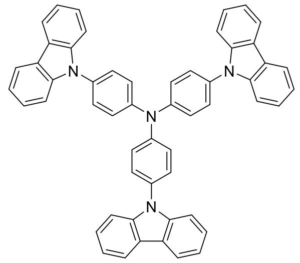

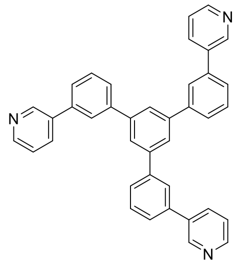

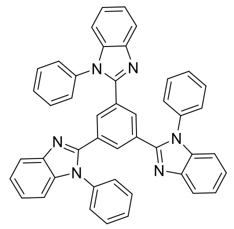

TCTA TmPyPB TPBi

Supplementary Figure 9. The chemical structures of organic materials used in OLEDs fabrication.

Section 1. Screening of the host material

We have tested various host materials in the very first step and fabricated doping films at a fixed doping concentration of $EuBr_2$-N_8. These films are highly sensitive to air as expected. Hence, it would be impossible for us to measure the accurate PLQY of each film. Shown below are the pictures of doping films with different host materials under excitation of 365 nm in glovebox. Although PLQY values are not accessible, it's obvious that TAPC (film **6**) and m-MTDATA (film **9**) exhibit the best performances. The PL spectra of film 1, 6, 8 and 9 are shown in Supplementary Fig. 11. The rest films are too weakly emissive to be measured. It is worth noting that the luminescent intensity is not proportional to the actual PLQY because the light absorbance may be different.

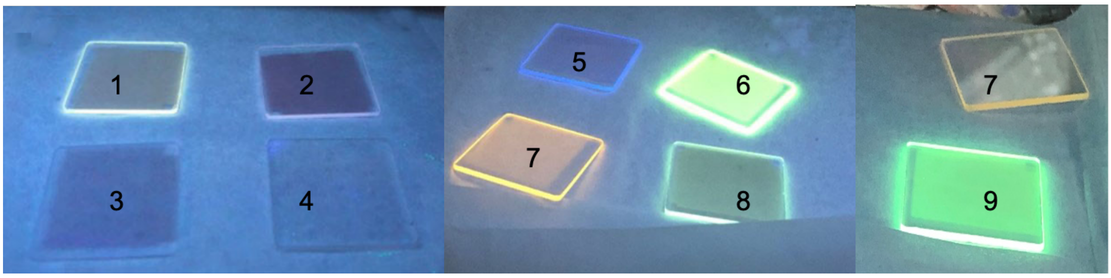

Supplementary Figure 10. The $EuBr_2$-N_8 pure film and $EuBr_2$-N_8 doping films using different host materials. Pictures were taken under UV excitation inside the glovebox. **1**: TCTA, **2**: CBP, **3**: TPBi, **4**: DPEPO, **5**: NPB, **6**: TAPC, **7**: $EuBr_2$-N_8: pure film, **8**: DIC-TRZ, **9**: m-MTDATA.

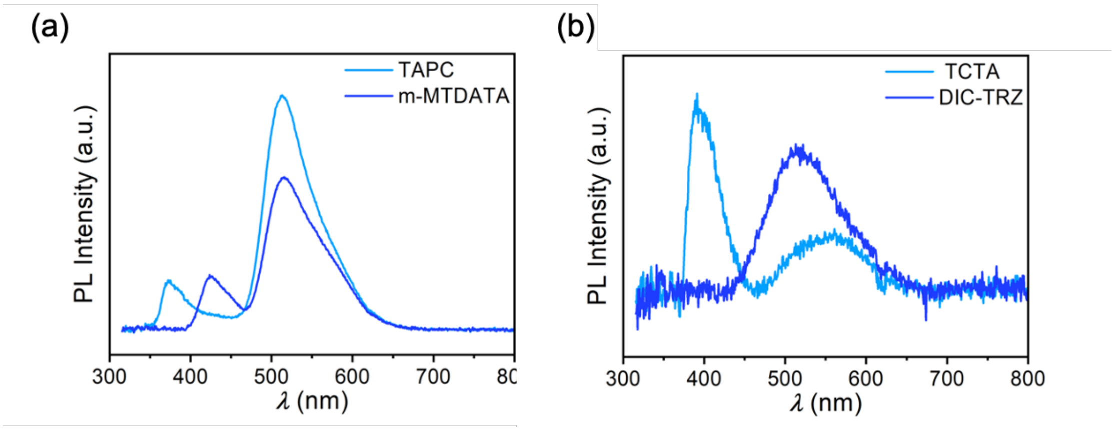

Supplementary Figure 11. The PL spectra of different doping films.

Then, different host materials m-MTDATA and TAPC were tested in devices **A1-A2**. The device **A1** showed the highest EQE of 7.6% with the smaller efficiency roll-off. Therefore, the m-MTDATA was selected for further optimization. The device structures are listed below (the changed factor and emissive materials are bolded):

A1: ITO/MoO_3 (2 nm)/m-MTDATA (40 nm)/**$EuBr_2$-N_8:m-MTDATA** (10 wt%, 20 nm)/TmPyPB (40 nm)/LiF (0.7 nm)/Al (100 nm)

A2: ITO/MoO_3 (2 nm)/m-MTDATA (40 nm)/**$EuBr_2$-N_8:TAPC** (10 wt%, 20 nm)/TmPyPB (40 nm)/LiF (0.7 nm)/Al (100 nm)

Supplementary Table 3. The key parameters of devices in Section 1.

Devices	V_{on}[a)] [V]	EQE_{max}[b)] [%]	EQE[c)] (100)	EQE[d)] (1000)	CE_{max}[e)] [cd A^{-1}]	L_{max}[f)] [cd m^{-2}]
A1	4.2	7.6	4.1	1.8	24	4254
A2	5.6	4.5	2.3	\	14.3	895

[a)] Turn-on voltage, is taken as a reference point at which the luminance is 1 cd m^{-2}, [b)] Maximum EQE, [c)] EQE at the luminance of 100 cd m^{-2}, [d)] EQE at the luminance of 1000 cd m^{-2}, [e)] Maximum current efficiency, [f)] Maximum luminance before failure.

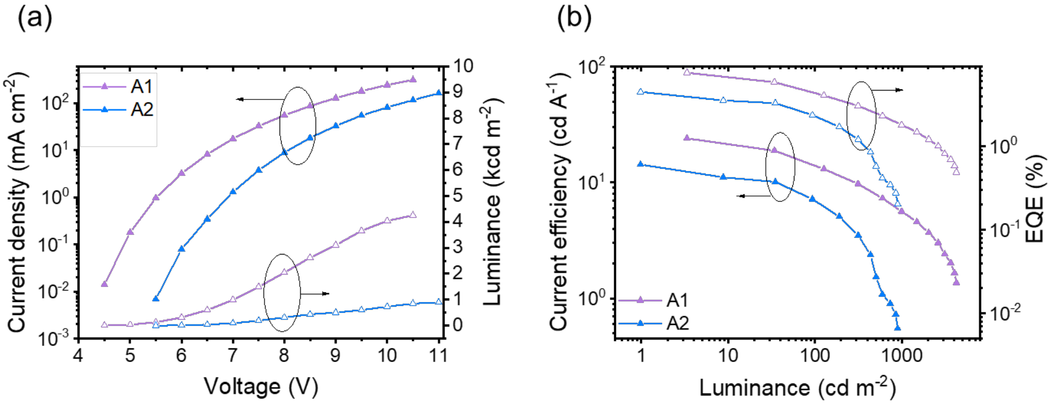

Supplementary Figure 12. (a) Current density-voltage-luminance (J-V-L) curves and (b) current efficiency-luminance- external quantum efficiency (CE-L-EQE) curves of devices **A1** and **A2.**

Section 2. Screening of the ETL & HTL materials

Different ETL materials varying from TmPyPB, DPEPO, Bphen, TPBi and their combination were designed in devices **A3-A10**. It was found that the device **A10** exhibited the best EQE of 12.4% with Bphen. However, **A10** device's efficiency roll-off was serious, which can be effectively improved through the introduction of TSPO1. As for HTL materials, we also tested m-MTDATA, TCTA, TAPC and so on. The device **A12** with TAPC showed the best behavior with the small efficiency roll-off and the maximum luminance. Considering that the simple structure is helpful to simplify the further optimization, we temporarily chose the combination of TAPC and Bphen.

A3: ITO/MoO_3 (2 nm)/**m-MTDATA (40 nm)**/$EuBr_2$-N_8:m-MTDATA (10 wt%, 20 nm)/**DPEPO (10 nm)**/**TmPyPB (30 nm)**/LiF (0.7 nm)/Al (100 nm)
A4: ITO/MoO_3 (2 nm)/**m-MTDATA (40 nm)**/$EuBr_2$-N_8:m-MTDATA (10 wt%, 20 nm)/**DPEPO (40 nm)**/LiF (0.7 nm)/Al (100 nm)
A5: ITO/MoO_3 (2 nm)/**m-MTDATA (40 nm)**/$EuBr_2$-N_8:m-MTDATA (10 wt%, 20 nm)/**DPEPO (10 nm)**/**TPBi (30 nm)**/LiF (0.7 nm)/Al (100 nm)
A6: ITO/MoO_3 (2 nm)/**m-MTDATA (40 nm)**/$EuBr_2$-N_8:m-MTDATA (10 wt%, 20 nm)/**TPBi (40 nm)**/LiF (0.7 nm)/Al (100 nm)
A7: ITO/MoO_3 (2 nm)/**m-MTDATA (40 nm)**/$EuBr_2$-N_8:m-MTDATA (10 wt%, 20 nm)/**TSPO1 (40 nm)**/LiF (0.7 nm)/Al (100 nm)
A8: ITO/MoO_3 (2 nm)/**m-MTDATA (40 nm)**/$EuBr_2$-N_8:m-MTDATA (10 wt%, 20 nm)/**TSPO1 (10 nm)**/**Bphen (30 nm)**/LiF (0.7 nm)/Al (100 nm)
A9: ITO/MoO_3 (2 nm)/**m-MTDATA (40 nm)**/$EuBr_2$-N_8:m-MTDATA (10 wt%, 20 nm)/**TSPO1 (10 nm)**/**TPBi (30 nm)**/LiF (0.7 nm)/Al (100 nm)
A10: ITO/MoO_3 (2 nm)/**m-MTDATA (40 nm)**/$EuBr_2$-N_8:m-MTDATA (10 wt%, 20 nm)/**Bphen (40 nm)**/LiF (0.7 nm)/Al (100 nm)
A11: ITO/MoO_3 (2 nm)/**TCTA (40 nm)**/$EuBr_2$-N_8:m-MTDATA (10 wt%, 20 nm)/**Bphen (40 nm)**/LiF (0.7 nm)/Al (100 nm)
A12: ITO/MoO_3 (2 nm)/**TAPC (40 nm)**/$EuBr_2$-N_8:m-MTDATA (10 wt%, 20 nm)/**Bphen (40 nm)**/LiF (0.7 nm)/Al (100 nm)

Supplementary Table 4. The key parameters of devices in Section 2.

Devices	V_{on} [V]	EQE_{max} [%]	EQE (100)	EQE (1000)	CE_{max} [cd A^{-1}]	L_{max} [cd m^{-2}]
A3	4.6	6.2	4.1	2.1	19.9	4494
A4	5.6	6.7	4.2	1.7	21.6	3107
A5	5.1	7.9	4.6	2.3	25.4	4474
A6	4.8	10.4	5.2	2.0	32.4	4536
A7	4.2	6.6	4.9	2.3	21.3	4251
A8	4.3	8.5	6.0	2.8	27.8	5253
A9	5.1	7.1	5.9	2.4	23.1	4432
A10	4.7	12.4	6.4	2.0	39.9	4059
A11	5.7	2.3	1.0	0.4	6.9	1631
A12	5.1	11.8	7.8	4.8	38.3	6652

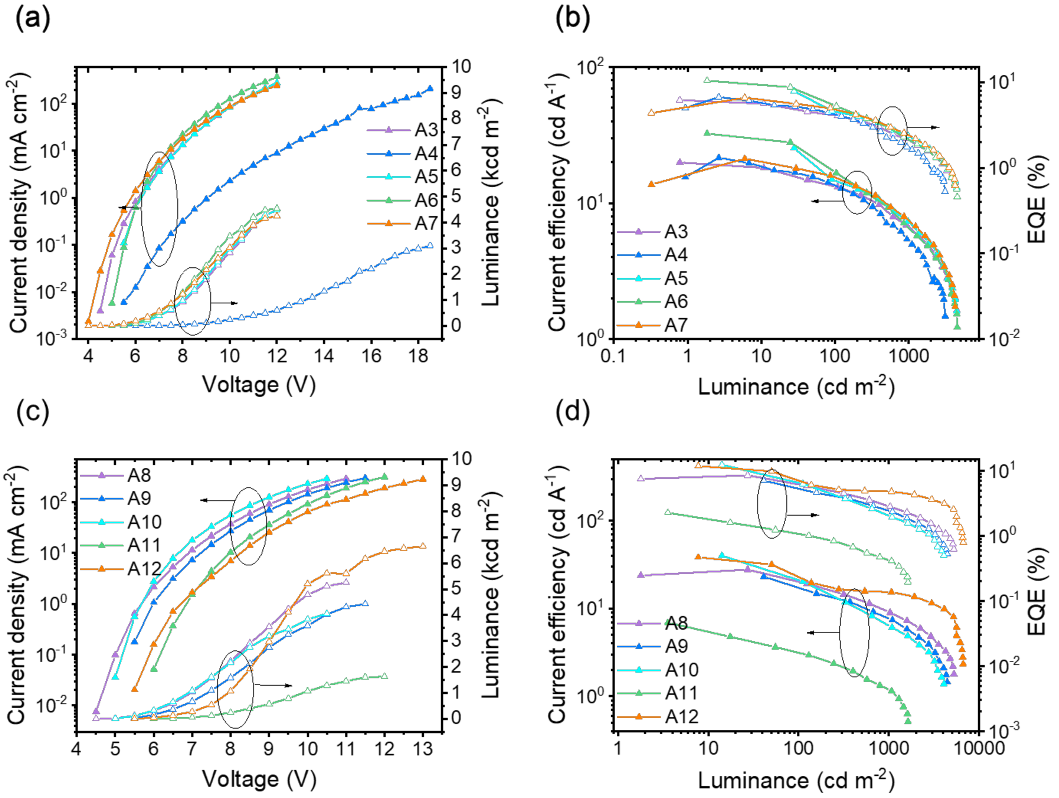

Supplementary Figure 13. (a) Current density-voltage-luminance (J-V-L) curves, and (b) current efficiency-luminance-external quantum efficiency (CE-L-EQE) curves of devices **A3-A7**. (c) Current density-voltage-luminance (J-V-L) curves, and (d) current efficiency-luminance-external quantum efficiency (CE-L-EQE) curves of devices **A8-A12**.

Section 3. Optimization of the thickness of emission layer

The thicknesses of EML varying among 15, 25, and 30 nm are tested in devices **A20-A22**. As the thickness of EML increases, the turn-on voltage increases significantly. In general, the best thickness is found to be 25 nm in **A22**.

A13: ITO/MoO_3 (2 nm)/TAPC (40 nm)/$EuBr_2$-N_8:m-MTDATA (~10 wt%, **15 nm**)/Bphen (40 nm)/LiF (0.7 nm)/Al (100 nm)
A14: ITO/MoO_3 (2 nm)/TAPC (40 nm)/$EuBr_2$-N_8:m-MTDATA (~10 wt%, **25 nm**)/Bphen (40 nm)/LiF (0.7 nm)/Al (100 nm)
A15: ITO/MoO_3 (2 nm)/TAPC (40 nm)/$EuBr_2$-N_8:m-MTDATA (~10 wt%, **30 nm**)/Bphen (40 nm)/LiF (0.7 nm)/Al (100 nm)

Supplementary Table 5. The key parameters of devices in Section 3.

Devices	V_{on} [V]	EQE_{max} [%]	EQE (1000)	EQE (4000)	CE_{max} [cd A^{-1}]	L_{max} [cd m^{-2}]
A13	4.7	8.8	4.3	2.5	28.5	5950
A14	6.1	14.4	5.3	3.4	47.1	7348
A15	7.0	8.1	5.9	3.6	26.7	7501

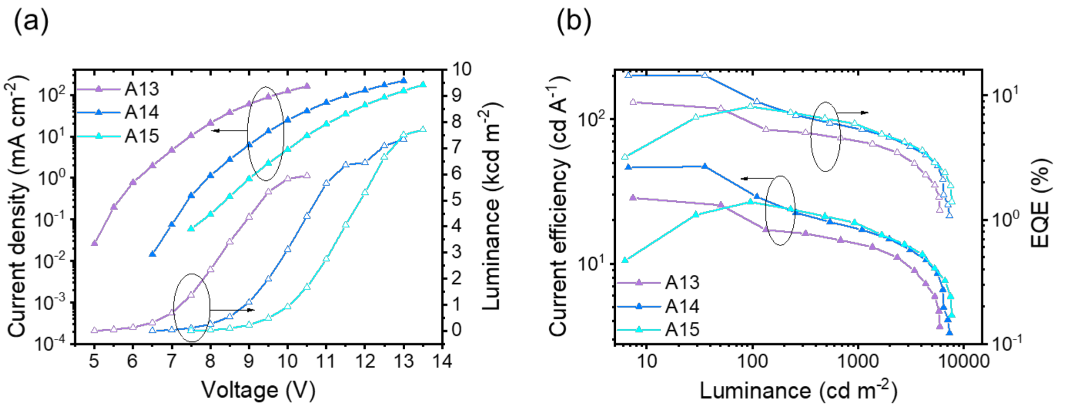

Supplementary Figure 14. (a) Current density-voltage-luminance (J-V-L) curves, and (b) current efficiency-luminance-external quantum efficiency (CE-L-EQE) curves of devices **A13-A15**.

Section 4. Final minor adjustments of $EuBr_2$-N_8 device

At last, we made some minor adjustments of the $EuBr_2$-N_8 device. After inserting the NPB and TSPO1 layers, the maximum EQE and luminance have been further improved with smaller efficiency roll-off.

A16: ITO/MoO_3 (2 nm)/**NPB (50 nm)**/TAPC (10 nm)/$EuBr_2$-N_8:m-MTDATA (~10 wt%, 25 nm)/**TSPO1 (10 nm)**/Bphen (30 nm)/LiF (0.7 nm)/Al (100 nm)

Supplementary Table 6. The key parameters of devices in Section 4.

Devices	V_{on} [V]	EQE_{max} [%]	EQE (1000)	EQE (4000)	CE_{max} [cd A^{-1}]	L_{max} [cd m^{-2}]
A16	6.2	15.5	7.0	5.0	52.8	10200

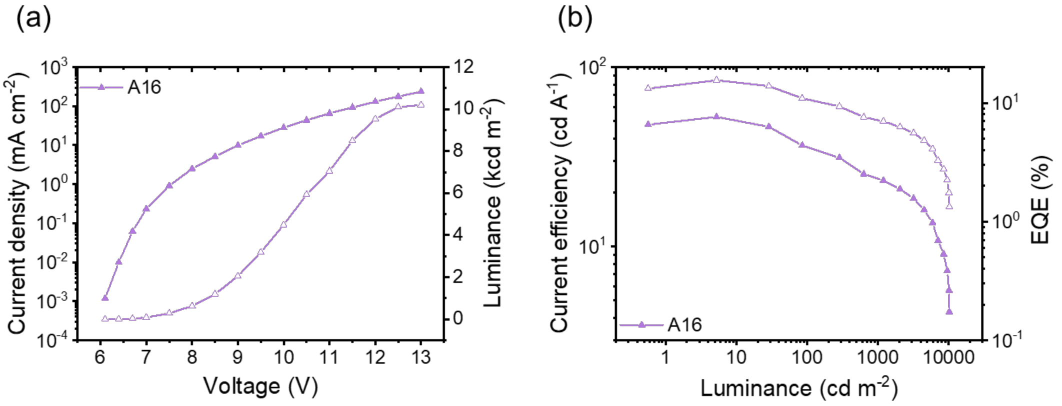

Supplementary Figure 15. (a) Current density-voltage-luminance (J-V-L) curve, and (b) current efficiency-luminance-external quantum efficiency (CE-L-EQE) curve of device **A16**.

Section 5. Optimization of the doping concentration in EuI_2-N_8 devices

Considering the maximum EQE, luminance, the turn-on voltage and the efficiency roll-off in these devices, we chose 7 wt% as the best doping concentration for EuI_2-N_8 devices.

B1: ITO/MoO_3 (2 nm)/NPB (50 nm)/TAPC (10 nm)/EuI_2-N_8:m-MTDATA (**5 wt%**, 25 nm)/TSPO1 (10 nm)/Bphen (30 nm)/LiF (0.7 nm)/Al (100 nm)
B2: ITO/MoO_3 (2 nm)/NPB (50 nm)/TAPC (10 nm)/EuI_2-N_8:m-MTDATA (**7 wt%**, 25 nm)/TSPO1 (10 nm)/Bphen (30 nm)/LiF (0.7 nm)/Al (100 nm)
B3: ITO/MoO_3 (2 nm)/NPB (50 nm)/TAPC (10 nm)/EuI_2-N_8:m-MTDATA (**10 wt%**, 25 nm)/TSPO1 (10 nm)/Bphen (30 nm)/LiF (0.7 nm)/Al (100 nm)
B4: ITO/MoO_3 (2 nm)/NPB (50 nm)/TAPC (10 nm)/EuI_2-N_8:m-MTDATA (**12 wt%**, 25 nm)/TSPO1 (10 nm)/Bphen (30 nm)/LiF (0.7 nm)/Al (100 nm)

Supplementary Table 7. The key parameters of devices in Section 5.

Devices	V_{on} [V]	EQE_{max} [%]	EQE (1000)	EQE (4000)	CE_{max} [cd A^{-1}]	L_{max} [cd m^{-2}]
B1	4.7	16.3	14.7	10.0	56.9	22980
B2	5.0	17.5	15.2	10.1	61.4	23470
B3	5.6	17.3	15.2	9.2	60.9	22220
B4	6.2	17.5	14.6	8.0	61.6	20270

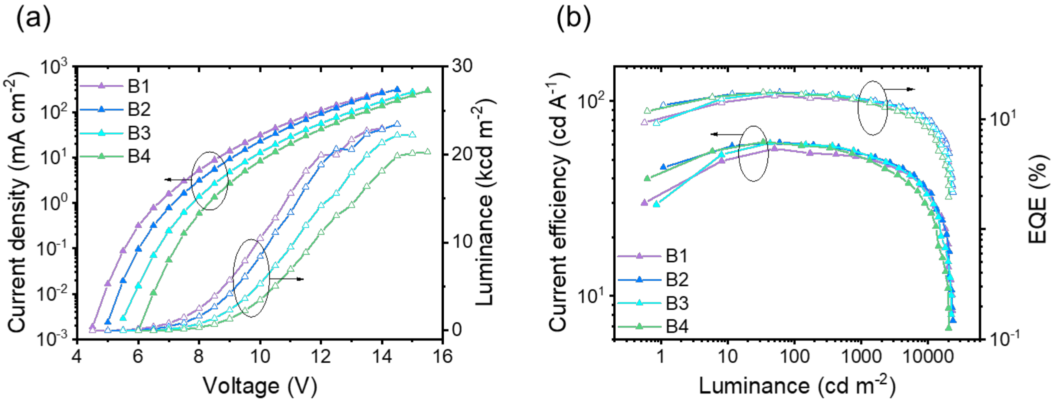

Supplementary Figure 16. (a) Current density-voltage-luminance (J-V-L) curves, and (b) current efficiency-luminance-external quantum efficiency (CE-L-EQE) curves of devices **B1-B4**.

Section 6. Optimization of the thickness of emission layer

Considering the maximum EQE, luminance and the efficiency roll-off of the devices, 25 nm is the best thickness.

B5: ITO/MoO_3 (2 nm)/NPB (50 nm)/TAPC (10 nm)/EuI_2-N_8:m-MTDATA (7 wt%, **15 nm**)/TSPO1 (10 nm)/Bphen (30 nm)/LiF (0.7 nm)/Al (100 nm)
B6: ITO/MoO_3 (2 nm)/NPB (50 nm)/TAPC (10 nm)/EuI_2-N_8:m-MTDATA (7 wt%, **20 nm**)/TSPO1 (10 nm)/Bphen (30 nm)/LiF (0.7 nm)/Al (100 nm)
B7: ITO/MoO_3 (2 nm)/NPB (50 nm)/TAPC (10 nm)/EuI_2-N_8:m-MTDATA (7 wt%, **25 nm**)/TSPO1 (10 nm)/Bphen (30 nm)/LiF (0.7 nm)/Al (100 nm)
B8: ITO/MoO_3 (2 nm)/NPB (50 nm)/TAPC (10 nm)/EuI_2-N_8:m-MTDATA (7 wt%, **30 nm**)/TSPO1 (10 nm)/Bphen (30 nm)/LiF (0.7 nm)/Al (100 nm)

Supplementary Table 8. The key parameters of devices in Section 6.

Devices	V_{on} [V]	EQE_{max} [%]	EQE (1000)	EQE (4000)	CE_{max} [cd A^{-1}]	L_{max} [cd m^{-2}]
B5	4.2	14.0	12.2	10.0	49.0	15970
B6	4.8	16.8	14.9	9.2	59.1	21810
B7	5.2	17.7	15.9	10.1	62.4	25470
B8	5.7	17.1	16.0	10.9	60.6	27180

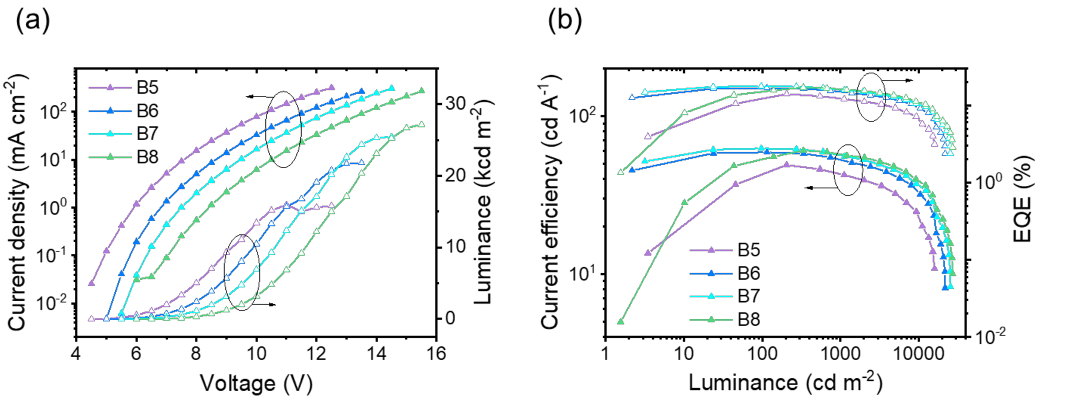

Supplementary Figure 17. (a) Current density-voltage-luminance (J-V-L) curves, and (b) current efficiency-luminance-external quantum efficiency (CE-L-EQE) curves of devices **B5-B8**.

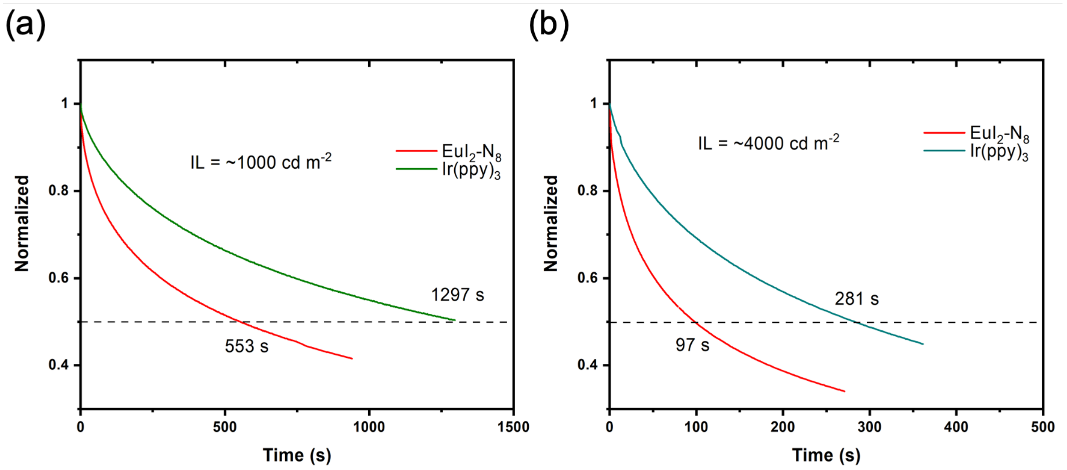

Supplementary Figure 18. The EL lifetime with fixed current density of (a) 2.5 mA/cm^2 (b) 10 mA/cm^2. The LT50 values are noted in the figures. The controlled device used Ir(ppy)$_3$ as emitters.

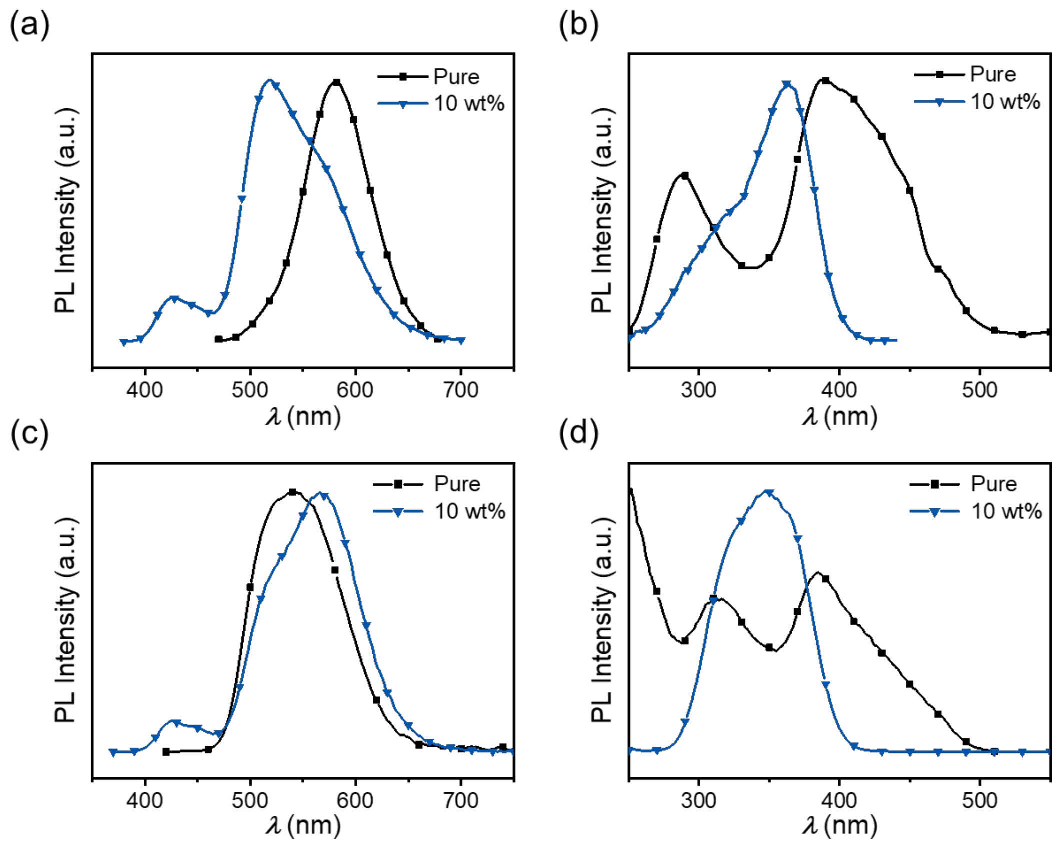

Supplementary Figure 19. (a) The emission spectra and (b) excitation spectra of $EuBr_2$-N_8:m-MTDATA (10 wt%) doping film and the $EuBr_2$-N_8 pure film. (c) The emission spectra and (d) excitation spectra of EuI_2-N_8:m-MTDATA (10 wt%) doping film and the EuI_2-N_8 pure film.

Supplementary References:

1. Neese, F. The ORCA program system. *Wiley Interdisciplinary Reviews: Computational Molecular Science*. **2**, 73-78 (2012).
2. Becke, A. Density-functional thermochemistry. III. The role of exact exchange. *J. Chem. Phys.* **98**, 5648-5652 (1993).
3. Lee, C. Yang, W. & Parr, R., Development of the Colle-Salvetti correlation-energy formula into a functional of the electron density. *Phys. Rev. B*. **37**, 785-789 (1988).
4. Vosko, S., Wilk, L. & Nusair, M. Accurate spin-dependent electron liquid correlation energies for local spin density calculations: a critical analysis. *Can. J. Phys.* **58**, 1200-1211 (1980).
5. Stephens, P., Devlin, F., Chabalowski, C. & Frisch, M. Ab Initio Calculation of Vibrational Absorption and Circular Dichroism Spectra Using Density Functional Force Fields. *J. Phys. Chem.* **98**, 11623-11627 (1994).
6. Dolg, M., Stoll, H., & Preuss, H. Energy-adjusted ab initio pseudopotentials for the rare earth elements. *J. Chem. Phys.* **90**, 1730-1734 (1989).
7. Weigend, F. & Ahlrichs, R. Balanced basis sets of split valence, triple zeta valence and quadruple zeta valence quality for H to Rn: Design and assessment of accuracy. *Phys. Chem. Chem. Phys.* **7**, 3297-3305 (2005).
8. Grimme, S., Ehrlich, S., & Goerigk, L. Effect of the damping function in dispersion corrected density functional theory. *J. Comput. Chem.* **32**, 1456-1465 (2011).
9. Grimme, S. Accurate description of van der Waals complexes by density functional theory including empirical corrections. *J. Comput. Chem.* **25**, 1463-1473 (2004).
10. Grimme, S. Semiempirical GGA-type density functional constructed with a long-range dispersion correction. *J. Comput. Chem.* **27**, 1787-1799 (2006).
11. Grimme, S., Antony, J., Ehrlich, S. & Krieg, H. A consistent and accurate ab initio parametrization of density functional dispersion correction (DFT-D) for the 94 elements H-Pu. *J. Chem. Phys.* **132**, 154104 (2010).
12. Schäfer, A., Horn, H. & Ahlrichs, R. Fully optimized contracted Gaussian basis sets for atoms Li to Kr. *J. Chem. Phys.* **97**, 2571-2577 (1992).
13. Neese, F., Wennmohs, F., Hansen, A. & Becker, U. Efficient, approximate and parallel Hartree–Fock and hybrid DFT calculations. A 'chain-of-spheres' algorithm for the Hartree–Fock exchange. *Chem. Phys.* **356**, 98-109 (2009).
14. Weigend, F. Accurate Coulomb-fitting basis sets for H to Rn. *Phys. Chem. Chem. Phys.* **8**, 1057-1065 (2006).
